# Magnetorheological Elastomer Stress Relaxation Behaviour during Compression: Experiment and Modelling

**DOI:** 10.3390/ma13214795

**Published:** 2020-10-27

**Authors:** Mateusz Kukla, Łukasz Warguła, Krzysztof Talaśka, Dominik Wojtkowiak

**Affiliations:** Institute of Machine Design, Faculty of Mechanical Engineering, Poznan University of Technology, Piotrowo 3, PL-60965 Poznan, Poland; lukasz.wargula@put.poznan.pl (Ł.W.); krzysztof.talaska@put.poznan.pl (K.T.); dominik.wojtkowiak@put.poznan.pl (D.W.)

**Keywords:** mechanical properties of advanced materials, magneto-rheological (MR), elastomer, stress relaxation, mathematical model

## Abstract

Materials characterized by magnetorheological properties are non-classic engineering materials. A significant increase in the interest of the scientific community about this group of materials could be observed over the recent years. The results of research presented in this article are oriented on the examination of the said materials’ mechanical properties. Stress relaxation tests were carried out on cylindrical samples of magnetorheological elastomers loaded with compressive stress, for various values of magnetic induction ($B_{1}$ = 0 mT, $B_{2}$ = 32 mT, $B_{3}$ = 48 mT, and $B_{4}$ = 64 mT) and temperature ($T_{1}$ = 25 °C, $T_{2}$ = 30 °C, and $T_{3}$ = 40 °C). The results of these tests indicate that the stiffness of the examined samples increased along with the increase of magnetic field induction, and decreased along with the increase of temperature. On this basis, it has been determined that: the biggest stress amplitude change, caused by the influence of magnetic field, was $\sigma_{0}^{\Delta B}$ = 12.7%, and the biggest stress amplitude change, caused by the influence of temperature, was $\sigma_{0}^{\Delta T}$ = 11.3%. As a result of applying a mathematical model, it was indicated that the stress relaxation in the examined magnetorheological elastomer, for the adopted time range (t = 3600 s), had a hyperbolic decline nature. The collected test results point to the examined materials being characterized by extensive rheological properties, which leads to the conclusion that it is necessary to conduct further tests in this area.

## 1. Introduction

Magnetorheological elastomers (MRE) are a group of composite materials, the mechanical properties of which change under the influence of an applied magnetic field [1,2]. This change occurs in a time range counting up to several milliseconds, and is fully reversible [3,4]. They combine materials with various properties: elastic polymers, and metals with good magnetic properties. Magnetorheological elastomers are often referred to as solid state magnetorheological fluids [5]. However, certain differences exist between these two material groups. The most important difference is that magnetic particles in elastomer composites have a very limited ability to move in the matrix [6,7,8]. The change of their position is in practice possible only through elastic deformation of the matrix solid material. Due to the differences in structure, magnetorheological elastomers operate in a load range different from fluids, i.e., below the yield point [9]. These two types of materials are complementary rather than competitive, because of the nature of their work.

Due to the unique properties of magnetorheological elastomers, many researchers focus their attention on the behavior of the elastomers in a magnetic field. The most frequently conducted tests concern the identification of the material properties spectrum related to the composite being loaded with shear stress [10]. However, polymer matrix-based composites from this group can also transfer different types of loads. Therefore, it is possible to indicate applications not only in torsion [11,12,13], but also in the compression mode [14]. Thus, it is quite clear that the test of the composite materials’ mechanical properties, for example, during compression, is also extremely important [15,16,17].

Designing devices utilizing magnetorheological elastomers in practical applications requires the repeatability of their behavior, and the upkeep of set settings resulting from their properties. The long-term and fault-free operation of devices using the discussed materials requires a thorough recognition of their properties, both in relation to time and temperature. Elastomer materials are characterized by the decline of their mechanical properties with the passage of time. Such a process is known as aging [18,19]. Additionally, the mechanical properties of polymers in general are strongly depend on temperature [20,21]. The tests of Wan et al. from 2018 show that the glass transition of magnetorheological samples, manufactured on the basis of silicone rubber, at uniaxial compression occurs at the temperature of approximately 50 °C. The value of storage modulus points to two different trends, occurring along with temperature changes: at first it rapidly declines and then slightly increases, or maintains a constant value, as the temperature increases [22]. Systems that will use MRE can heat up as a result of heat radiating from co-operating devices, e.g., drive units [23], gearboxes [24], or working mechanisms [25]. Energy dissipation (resulting from regular operation) also increases the temperature of polymer materials, due to the occurrence of internal friction [26]. Moreover, they are characterized by complex rheological properties that characterize, among other things, creep and stress relaxation phenomena [27]. For that reason, it is justified to conduct research aimed at examination of the aforementioned properties. Research related to the issue of stress relaxation can also be found in the literature [28,29,30].

The purpose of the presented research was to determine the impact of magnetic field induction (B), temperature (T), and time (t) on the mechanical properties of magnetorheological elastomers subjected to uniaxial compressive stress. The analysis of the available literature regarding magnetorheological elastomers and polymers served as a basis for the hypothesis that the increase of stiffness in examined samples will depend on the increase of parameters characterizing the magnetic field, and that it will be inversely dependent on the increase of temperature.

## 2. Materials and Methods

### 2.1. Sample Preparation

**Figure 1:**
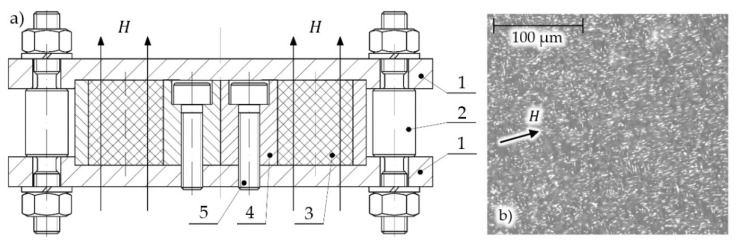
(**a**) Magnetorheological elastomer (MRE) sample manufacturing mold diagram; 1: mold covers, 2: cover fixing elements, 3: magnetorheological elastomer, 4: mold, 5: mold fixing elements; (**b**) microscope photo of internal sample structure.

As part of the research, testes was undertaken on the stress relaxation of the studied magnetorheological elastomers. As part of a single test, the MRE sample was loaded (compressed) until the defined strain value was obtained. Then the strain value was maintained for a set amount of time. Elastomer materials (and thus also their composites) are characterized by viscoelastic properties, and for this reason it is expected that stress in a sample loaded in such way will gradually decrease. In other words, the stress relaxation phenomenon will occur. Samples were made as a result of combining the Sika-brand Biresin U1404 elastomer, and carbonyl iron powder with a particle diameter between 6 and 9 µm. The content of ferromagnetic particles in the composite was 33 vol%. The carbonyl iron powders are commonly used magnetic particles in the process of MRE preparation [31]. Specific components were combined through mechanical mixing, followed by degassing and placement in a mold designed specifically for this purpose. The diagram of the mold is shown in Figure 1a. The aluminum alloy mold (4) was located between two steel covers (1). Inside the mold were located channels that gave the magnetorheological elastomer (3) the required geometrical properties. The mold was fixed to the cover with the help of bolts (5). The tight closure of the mold by covers (1) was ensured by properly tensioned aluminum alloy bolts (2). During sample curing, the mold was subjected to a magnetic field along their axles. The appropriate selection of applied materials makes the magnetic flux close through the sample during its manufacturing. This occurs because the applied aluminum alloy and air have significantly lower magnetic permeability than the mold covers, and the magnetorheological composite itself. The created samples had the shape of cylinders, with a diameter, d = 20 mm, and a height, h = 20 mm. The curing of samples was carried out at room temperature and in the presence of a magnetic field, with the magnetic induction, B = 300 mT. The samples were manufactured in such an amount that each iteration could be conducted on a new sample; this eliminated the necessity of taking the history of sample loading into account. The microscope photos of the examined samples’ cross-sections are shown in Figure 1b; iron particles are presented as brighter fields.

### 2.2. Experiment Detalis

**Figure 2:**
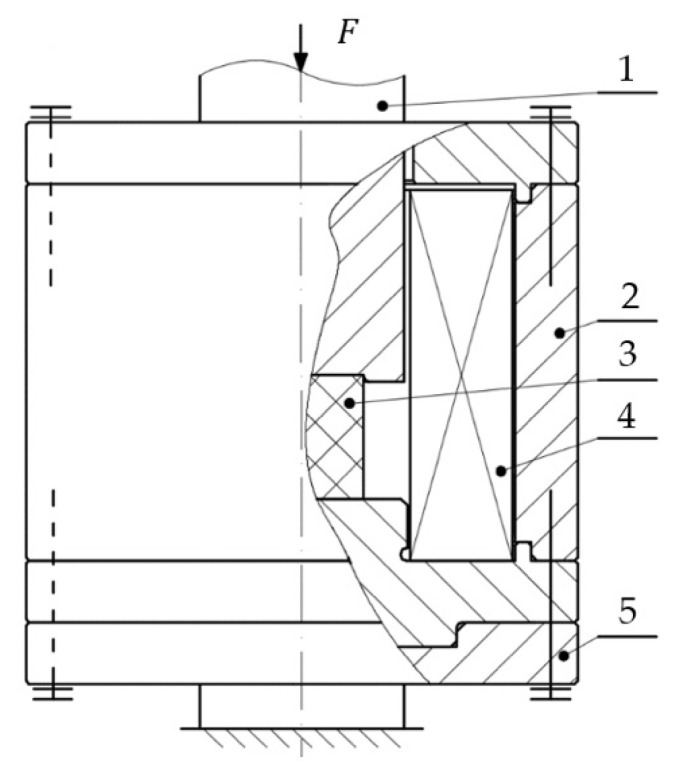
Test stand diagram; 1: element transferring compressive force, 2: housing, 3: examined sample, 4: coil, 5: base.

The tests were carried out on an MTS Insight universal testing machine. The measurement of force value was performed using an HBM-brand C9C 1 kN sensor. An HBM-brand WA 20 mm linear position sensor was used to determine the displacement value. The whole system co-operated with a Spider 8 measurement system connected to a PC. In addition, the system was equipped with a specially designed, original test stand, the purpose of which was to create a magnetic field with set parameters, in order to conduct further experiments. The system diagram is shown in Figure 2. The housing (2), as well as the covers, was made from a special iron alloy characterized by good magnetic properties. After machining, the housing elements were subjected to annealing, in order to acquire uniform properties in their whole cross-section. Inside the housing was located an induction coil (4) along with the magnetorheological elastomer sample (3). The whole system was fixed to a base made from non-magnetic austenitic steel. This ensured the closure of magnetic flux through the examined sample.

**Figure 3:**
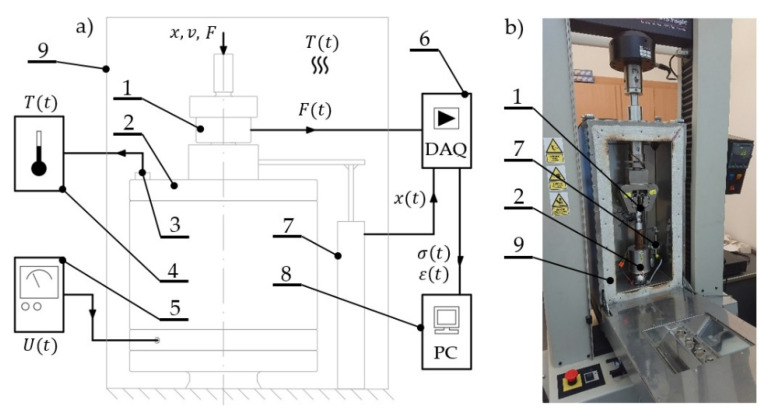
Measurement system: (**a**) diagram, (**b**) view: 1: force sensor, 2: measurement position, 3: temperature sensor, 4: temperature converter, 5: laboratory power supply unit, 6: measurement amplifier, 7: displacement sensor, 8: computer, 9: climate chamber.

The test stand was placed in a climate chamber dedicated to the utilized universal testing machine, which allowed controlling and measuring the temperature inside its working space. In addition, the control of the temperature value during experiments was carried out using a Seek Thermal-brand RevealPRO FF RQEAAX thermal vision camera. The samples were compressed to a set deformation amplitude, ε = 20%, with speed, v = 0.8 mm/s. For the conditions set in such a way, a sample relaxation was conducted in a time range equal to t = 3600 s, in which the variable parameters were constituted by magnetic induction values $B_{1}$ = 0 mT, $B_{2}$ = 32 mT, $B_{3}$ = 48 mT, and $B_{4}$ = 64 mT, and temperatures $T_{1}$ = 25 °C, $T_{2}$ = 30 °C, and $T_{3}$ = 40 °C. The measurement system diagram is shown in Figure 3a, while its view is shown in Figure 3b.

## 3. Results and Discussion

### 3.1. Experimental Research

**Figure 4:**
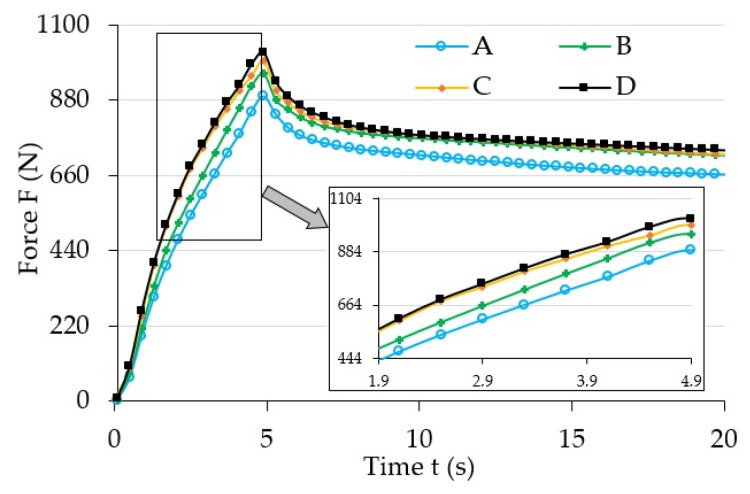
Selected test results for various magnetic field induction B values for temperature, and value T = 25 °C; A: 0 mT, B: 32 mT, C: 48 mT, D: 64 mT.

**Figure 5:**
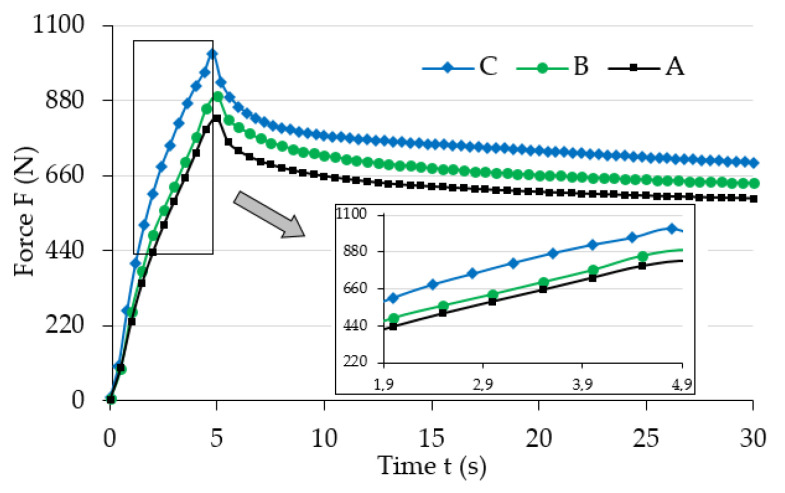
Selected test results for various temperature T values for magnetic induction with value B = 0 mT; temperature value: A: 25 °C, B: 30 °C, C: 40 °C.

**Figure 6:**
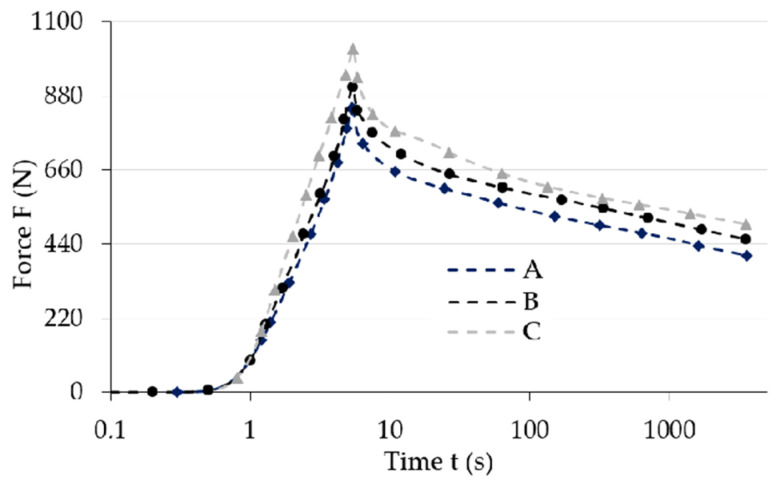
Selected test results for various temperature T values for magnetic induction with value B = 0 mT (in the logarithmic scale of time; for the whole duration of a single experiment); temperature value: A: 25 °C, B: 30 °C, C: 40 °C.

Figure 4 shows the initial range of sample load relaxation test results for various magnetic induction B values. Figure 5 shows the initial range of sample load relaxation test results for various magnetic induction t values, while Figure 6 shows the same results for the whole analyzed time range T.

The analysis of the results (Figure 4) allows noticing the increase of maximum force (strain) value for the examined samples that occurred along with the increase of magnetic field induction, B. It also had an influence on the progress of the stress relaxation process in the examined samples. The collected results suggest that the increase of magnetic field parameters caused the increase (extension) of the load relaxation time. Such a phenomena can be explained by the mutual influence of the magnetic field and ferromagnetic particles. During manufacturing they showed a tendency to group in columns, according to the direction of the applied magnetic field [32], due to magnetization. In addition, ferromagnetic particles aim to properly orient their easy magnetization directions in space, that is, in parallel to the line of magnetic flux. Their arrangement is the result of the system’s attempt to reach the state of minimum energy. In this position they will be fixed after the curing completion of the polymer matrix. Later deformation of such material requires a greater amount of energy, i.e., performing additional work (applying greater force), when compared to the deformation of magnetorheological elastomer not subjected to the magnetic field. This energy is used to relocate ferromagnetic particles from positions with minimum energy. The amount of required work to be supplied depends on the intensity of the applied magnetic field [33]. Thus, an apparent increase of mechanical properties, for example, the stiffness module, which are the function of magnetic field induction, will be observed when analyzing the properties of MRE samples on a macro-scale. The value of stiffness module has a direct impact on the progress of the discussed process in relation to both the maximum force (load) value, and stress relaxation time.

The analysis of results (Figure 5 and Figure 6) allows noticing the decline of maximum force (load) value for the examined samples that occurs along with the increase of temperature T. The stress relaxation phenomenon depends on temperature because the increase of temperature causes a proportional reduction of internal friction, which in consequence shortens the stress relaxation time. This phenomenon also depends on the polymer’s structure, and the type of network and bonds created in the vulcanizate. This is caused by the fact that the structure of elastomers has a macro-particle structure. The molecular chains that comprise the composite matrix have the ability to move in relation to each other. Therefore, materials from this group are characterized by the occurrence of mechanical hysteresis, which is one of few energy dissipation ability indicators. The increase of temperature results in the increased mobility of molecular chains and, as a consequence, the reduction of the load value necessary to deform the samples. Thus, a decline of mechanical properties, for example the stiffness module, which are the function of temperature, will be observed when analyzing the properties of MRE samples on a macro-scale.

**Figure 7:**
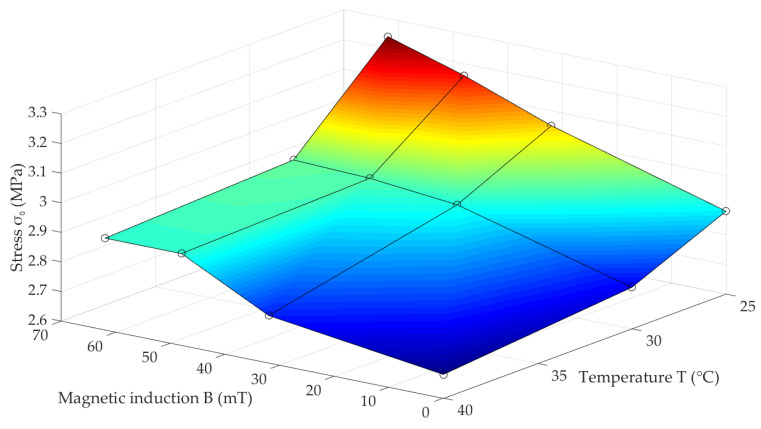
Stress amplitude ($\sigma_{0}$) dependency on magnetic field induction (B) and temperature (T) for the examined MRE samples.

The properties of a magnetorheological elastomer as a composite material strongly depend on its manufacturing method, and the materials used for this purpose [3]. Quite often, various non-magnetic additives for the polymer matrix material are used to change the MRE mechanical properties, while maintaining the fixed content of magnetic phase [34]. As a material group, elastomers are characterized by strongly developed rheological properties. For this reason, the influence of amplitude and deformation velocity on the MRE properties also cannot be omitted in general. These two values in the discussed experiment were fixed, and therefore their influence on the recorded results was not determined. This was taken into account when determining the maximum load value ($\sigma_{0}$) as a function of temperature (T) and magnetic field induction (B), which has been shown in Figure 7. This physical quantity carries the information about the stiffness of the examined composite, and can be used as an indicator of upcoming changes in mechanical properties. Depending on magnetic induction ($\sigma_{0}^{\Delta B}$), the values of relative stress amplitude increases were determined according to dependence (1):

(1) $$\sigma_{0}^{\Delta B}=\frac{\sigma_{0}^{Bmax}-\sigma_{0}^{Bmin}}{\sigma_{0}^{Bmin}}\cdot 100\%,$$

where $\sigma_{0}^{\Delta B}$ is a relative stress amplitude increase depending on magnetic induction, $\sigma_{0}^{Bmax}$ is the greatest strain amplitude value for the greatest magnetic induction value for a given temperature, and $\sigma_{0}^{Bmin}$ is the greatest strain amplitude value for the greatest magnetic induction value for a given temperature. The values of relative stress amplitude increase, depending on temperature $\sigma_{0}^{\Delta T}$, were determined in a similar way. The summary of the calculated results are presented in Table 1. As can be seen, the biggest stress amplitude change caused by the influence of the magnetic field was $\sigma_{0}^{\Delta B}$ = 12.7%, and the biggest stress amplitude change caused by the influence of temperature was $\sigma_{0}^{\Delta T}$ = 11.3%.

### 3.2. Mathematical Model

The initial attempts, focused on the application of a standard rheological model in the form of an Equation (2):

(2) $$\sigma \left(t\right)=E_{2}\varepsilon_{0}+E_{1}\varepsilon_{0}e^{-\frac{E_{1}}{\eta }t},$$

where $E_{1}$ and $E_{2}$ are elasticity moduli, $\varepsilon_{0}$ is strain amplitude, η is viscosity, and t is time, gave unsatisfactory results for the mathematical description of the examined composites’ properties. Therefore, a decision to apply a model presented in [35] was made. Its authors have laid out mathematical relationships intended for the description of stress relaxation phenomenon in the form of an Equation (3):

(3) $$\frac{1}{\sigma^{m-1}\left(t\right)}=\frac{1}{\sigma_{0}^{m-1}}\left[1+\left(m-1\right)\frac{E}{\eta }t\right],$$

where m is steady-state creep exponent and $\sigma_{0}$ is stress amplitude. After simple transformations, Equation (3) can be shown in the form of a dependency (4):

(4) $$\sigma \left(t\right)=\sigma_{0}{\left[1+\left(m-1\right)\frac{E}{\eta }t\right]}^{-\frac{1}{m-1}}.$$

In order to simplify the notation of Equation (4), functions (5) can be substituted:

(5) $$a=\frac{\eta }{E}\;\mathrm{and}\;b=\left(m-1\right)\;\mathrm{as}\;\mathrm{well}\;\mathrm{as}\;\sigma_{0}=q,$$

where a is load extenuating rate of the material, and b is a function of m (steady-state creep exponent). As a result, the final form of the equation acquired is given as (6):

(6) $$\sigma \left(t\right)=q{\left[1+\frac{b}{a}t\right]}^{-\frac{1}{b}}.$$

The analysis of Equations (2) and (6) allows us to draw a conclusion that both equations allow modeling a decreasing tendency. The value of stress ($\sigma \left(t\right)$) declines along with the increase of time (t) in each one of them. However, from the mathematical point of view Equation (2) models an exponential decline, while dependency (6) has a hyperbolic decline nature. For this reason, it seems that this is significantly better to fit the data recorded during experiments.

**Figure 8:**
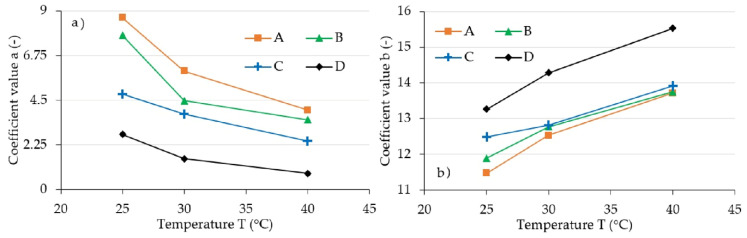
Set values of coefficients, (**a**) *a* and (**b**) *b* coefficients for various magnetic induction values; A: 64 mT, B: 48 mT, C: 32 mT, D: 0 mT.

**Figure 9:**
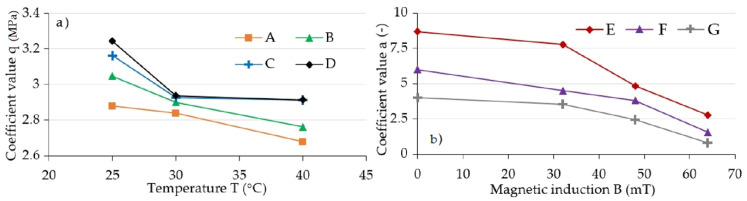
Set values of coefficients, (**a**) q for various magnetic induction values: A: 64 mT, B: 48 mT, C: 32 mT, D: 0 mT, (**b**) for various temperature values: E: 25 °C, F: 30 °C, G: 40 °C.

**Figure 10:**
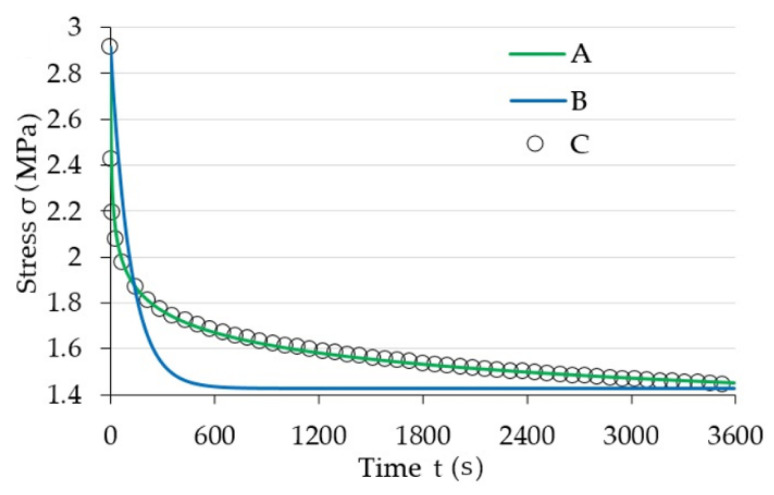
Summary of modelling results; A: rheological model acc. to [35], B: standard rheological model, C: measurement data.

This observation served as a basis for conducting numeric calculations aimed at determining a pair of Equation (6) a and b parameters in a way that the acquired curve allows for fitting the collected measurement data as accurately as possible. The procedure used during this activity was based on the least squares method. Parameter q corresponds to the stress amplitude ($\sigma_{0}$), and its value was read directly from experimental data. The summary of acquired characteristics in magnetic induction function is shown in Figure 8. For illustrative purposes, a characteristic that shows the dependency of factor a on temperature T was additionally included in Figure 9. The summary of results acquired, due to conducting an experiment, as a result of applying a model described with Equation (2), and in accordance with model (6) are shown in Figure 10. As can be seen, the latter is characterized by a significantly better quality of rendering set values.

According to the authors of model (2) [35], the value of parameter a expresses the load extenuating rate of the material. The value m, which defines parameter b, is the steady-state creep exponent. Parameter q is the straightforward stress amplitude ($\sigma_{0}$), which results from the greatest force value recorded in the experiment. The analysis of the included figures allows drawing a conclusion that the parameter a and q values decline with the temperature increase, while the value of parameter b increases. This is the effect of the previously described change of composite matrix material property, which is the result of internal friction change. The impact of magnetic field expressed through magnetic induction (B) results in the increase of parameter b and q values. This results from the internal structure of magnetorheological elastomer, and thus from the mutual influence of the magnetic field and the ferromagnetic particles of which the said composite consists.

The tests of magnetorheological elastomer properties in the range between 25 °C and 40 °C allow drawing conclusions similar to those drawn during tests of these materials by the Wan et al. team, in 2018, that elastomers in this temperature range are characterized by a significant loss of mechanical properties, along with temperature increase [22].

## 4. Conclusions

The results of both our own tests, and analysis of the available literature, allow us to declare that magnetorheological elastomers are materials with exceptionally complex properties. The properties of the manufactured composite depend on numerous factors. In the first order, these are: the applied materials of the components, and production methods. The further change of properties is related to the influence caused on ferromagnetic particles included in the composite matrix material by magnetic field. The scale of observed changes also depends on the applied load state, as polymer materials can withstand various types of stress, such as, compression, shearing, or bending, as well as any combinations of these. The test results also point to the complex rheological properties of magnetorheological elastomers. As a result, the load relaxation and creeping phenomena also carry some significance. Therefore, the important role in the analysis of materials from this group is taken not only by load and deformation values, but also by the dynamics of their changes in time. The impact of temperature is also non-negligible, because it has a crucial meaning for the properties of the composite matrix material. In general, the MRE loading history should also be taken into account, because the composite matrix material and ferromagnetic material contained in it are characterized by intermolecular interactions. The issue is additionally complicated by the overlap, or mutual influence, of several physical phenomena. For example, the time, in which the deformation can develop, is proportionally reduced along with the increase of strain velocity. Thus, in the context of stress relaxation time, for certain value ranges, temperature reduction results in changes with a similar nature to increasing strain velocity, and vice versa. Increasing the strain velocity causes the increase of force necessary to deform the elastomer, which is the result, and confirmation, of viscoelastic properties of the material. The next example is the mutual relation of mechanical and magnetic interactions. The compressive stress causes the MRE deformation, which results in change of distance between ferromagnetic material particles. Their mutual interaction, caused by the magnetic field, depends, inter alia, on their mutual position (distance between the particles). Therefore, in this context it is possible to show a mutual dependency between two different interactions.

To sum up, it should be concluded that magnetorheological elastomers in general are materials with complex properties. Further tests on the composites from this group, as well as the mutual relations between physical interactions related to them, are necessary

## Figures and Tables

**Table 1:** Summary of calculated stress amplitude values ($\sigma_{0}$).

		Magnetic Induction B (mT)				$\sigma_{0}^{\Delta B}\;(\%)$
		0	32	48	64	
		Stress Amplitude $\sigma_{0}$ (MPa)				
Temperature T (°C)	25	2.88	3.05	3.16	3.24	12.7
	30	2.74	2.90	2.93	2.94	7.2
	40	2.68	2.76	2.91	2.92	8.8
$\sigma_{0}^{\Delta T}$ (%)		7.5	10.3	8.6	11.3	
